# Comparison of High-Performance Liquid Chromatography with Sucrose Density Gradient Ultracentrifugation for the Quantification of Foot-and-Mouth Disease Vaccine Antigens

**DOI:** 10.3390/vaccines10050667

**Published:** 2022-04-22

**Authors:** Ah-Young Kim, Sun Young Park, Sang Hyun Park, Jae Young Kim, Jong Sook Jin, Eun-Sol Kim, Jong-Hyeon Park, Young-Joon Ko

**Affiliations:** Center for FMD Vaccine Research, Animal and Plant Quarantine Agency, Gyeonsangbuk-do, Gimcheon 39660, Korea; mochsha@korea.kr (A.-Y.K.); sun3730@korea.kr (S.Y.P.); shpark0205@korea.kr (S.H.P.); ivorikim@korea.kr (J.Y.K.); in75724@korea.kr (J.S.J.); kesol13@korea.kr (E.-S.K.); parkjhvet@korea.kr (J.-H.P.)

**Keywords:** foot-and-mouth disease virus (FMDV), quantification, sucrose density gradient ultracentrifugation (SDG), size-exclusion high-performance liquid chromatography (SE-HPLC), bovine enterovirus (BEV)

## Abstract

Foot-and-mouth disease (FMD) causes substantial economic losses in the livestock industry. The protective immunizing component of the FMD virus (FMDV) is a ribonucleoprotein particle with a sedimentation coefficient of 146S. Size-exclusion high-performance liquid chromatography (SE-HPLC) was introduced to replace sucrose density gradient ultracentrifugation (SDG), which is the gold standard for the quantification of FMDV 146S particles. SE-HPLC showed a pattern similar to that of SDG; however, the two methods resulted in different quantities for the same amount of 146S particles. This study aimed to identify the reason for this disparity and adjust the difference between the two methods by employing a standard material. While SE-HPLC displayed all the virus particles in the peak fraction by SDS-PAGE and Western blotting, the virus particles were widely dispersed in multiple fractions, including peak fractions in the SDG. To adjust the difference between the two methods, a stable surrogate virus, bovine enterovirus, was devised to draw a standard curve, and the gap was reduced to <10%. To our knowledge, this is the first report to provide experimental evidence on the difference between SDG and SE-HPLC for the quantification of FMDV particles.

## 1. Introduction

Foot-and-mouth disease (FMD) is a highly contagious vesicular disease that affects cloven-hoofed animals and frequently causes considerable economic losses to the livestock industry [1]. The FMD virus (FMDV), the causative agent of FMD, belongs to the *Aphthovirus* genus of the *Picornaviridae* family [2]. It has a positive-sense, single-stranded RNA genome that is translated into a polyprotein, which is further cleaved into structural and nonstructural proteins [3,4,5].

The viral capsid is assembled from 60 copies of each of the four structure proteins in a stepwise process [6]. First, one copy each of the proteins, VP0, VP3, and VP1, folds into a protomer, five copies of the protomer assemble into a pentamer, and twelve pentamers assemble into an icosahedral structure [7]. It is generally accepted that the protective immunizing component of FMDV is a ribonucleoprotein particle with a sedimentation coefficient of 146S [8]. The 146S particle is produced by the encapsidation of RNA within the 75S particle, followed by cleavage of VP0 to VP2 and VP4.

Sucrose density gradient ultracentrifugation (SDG) is the gold standard for the quantification of 146S particles [9]. It is widely used for fractioning specific macromolecules when a sample contains a mixture of different size macromolecules. By centrifugation, macromolecules in a sample, layered onto the surface of a linear sucrose gradient, can be separated because they sediment through the gradient at different rates depending on their size, shape, and density [10]. A peak corresponding to intact (146S) particles is detected close to the bottom of the gradient, because 146S particles are heavier than empty (75S) and dissociated (12S) particles [11]. However, this classic method involves several laborious processes, such as the preparation of sucrose gradient tubes, ultracentrifugation, and manual operation to measure the peak area. In addition, the number of concurrently treatable samples is limited. To address these drawbacks, size-exclusion high-performance liquid chromatography (SE-HPLC) was introduced to replace the classic SDG method [12,13,14]. SE-HPLC is a chromatographic method in which macromolecules can be separated by their size via filtration through a gel using high-performance liquid chromatography equipment [14]. Because smaller molecules remain within the pores of a gel for a longer time compared to a larger molecule, a peak corresponding to intact (146S) particles is detected earlier than dissociated (12S) particles and RNA [14,15].

Although SE-HPLC showed a similar pattern to the SDG for the quantification of 146S particles, previous studies reported that the two methods resulted in different quantities for the same amount of 146S particles [13,16]. This study aimed to identify the reason for the disparity between the two quantitation methods and provide instructions for adjusting the gap by employing a standard curve using a stable surrogate virus.

## 2. Materials and Methods

### 2.1. Viruses

Four FMDV strains were used in this study. FMDV O/Boeun/SKR/2017 (O BE, GenBank accession No. MG983730) and A/Yeoncheon/SKR/2017 (A YC, GenBank accession No. KY766148) were isolated by the Animal and Plant Quarantine Agency during FMD outbreaks in South Korea and adapted to BHK-21 suspension cells [17,18]. Recombinant A22 IRQ and O PA2 were constructed on the backbone of the O1 Manisa/Turkey/69 strain (GenBank accession No. AY593823) with the P1 region of A22 Iraq/24/64 (GenBank accession No. KY825717), and O PAK/44/2008 strain (GenBank accession No. GU384682) as described previously [19,20].

Bovine enterovirus (BEV) was acquired by transfecting BHK-21 suspension cells with pBLUBEV, a plasmid bearing the whole viral RNA sequence of BEV type 1 strain LCR4 (ATCC ^®^ number: VR-248TM), as described previously [21].

### 2.2. Preparation of Virus Samples

FMDV was inoculated into BHK-21 suspension cells at a multiplicity of infection (MOI) of 0.005 and incubated at 37 °C in a 5% CO_2_ shaking incubator at 110 rpm. Subsequently, the supernatant was harvested by centrifugation (4000× *g*, 20 min) at 16 h post-infection and inactivated by the addition of 3 mM binary ethylenimine (BEI; Sigma-Aldrich, St. Louis, MO, USA). The supernatant was then incubated in a shaking incubator at 26 °C for 24 h. Residual BEI was quenched using 2% sodium thiosulfate (Daejung Chemicals, Siheung-si, Korea). The inactivated virus was concentrated by mixing with a final concentration of 7.5% (*w*/*v*) PEG 6000 (Sigma-Aldrich) and 0.5 M NaCl (Sigma-Aldrich). The precipitate was obtained by centrifugation (10,000× *g* for 30 min), resuspended in 1 mL of Tris-KCl (TK) buffer (pH 7.6), and centrifuged (10,000× *g* for 10 min) to collect the supernatant. The sample solution was then layered onto 15–45% sucrose density gradients and ultracentrifuged again at 100,000× *g* for 4 h at 4 °C using an SW41Ti rotor (Beckman Coulter, Brea, CA, USA). Ultracentrifuged samples were fractionated using a continuous density gradient fractionator (Teledyne ISCO, Lincoln, NE, USA).

BEV was inoculated into BHK-21 suspension cells at an MOI of 0.01 and incubated at 37 °C in a 5% CO_2_ shaking incubator at 110 rpm for 24 h. After cell debris was removed by centrifugation (4000× *g*, 20 min), the virus concentration and purification were conducted in the same manner as that for FMDV.

### 2.3. 146S Particle Quantification with Fractionation

Quantification with fractionation of 146S particles was performed by either the SDG or SE-HPLC. For the SDG, 2 mL sample solution was layered onto 11 mL of 15–45% sucrose density gradient tubes and ultracentrifuged at 100,000× *g* for 4 h at 4 °C using an SW41Ti rotor (Beckman Coulter). The ultracentrifuged samples were fractionated using a continuous density gradient fractionator (Teledyne ISCO) and the absorbance of each fraction at 254 nm was recorded using the spectrophotometer component of the instrument. The area under the peak for specific fractions was measured to calculate the quantity of 146S particles (µg/mL), according to a previous study [9]. The peak fractions purified by SDG were concentrated by ultracentrifugation. Briefly, the band between the 30% and 35% sucrose layers was collected and ultracentrifuged at 100,000× *g* for 4 h. The resulting pellet was resuspended and dialyzed against Tris-KCl buffer (pH 7.6) to eliminate the residual sucrose at 4 °C. The concentrated peak fractions that contained purified 146S particles were applied to SDG and SE-HPLC to compare the quantitation values between the two methods. Purified 146S particles of FMDV were diluted serially in two-fold with Tris-KCl buffer (pH 7.6) from 1× to 1/32×, and quantified by each method on the same day, respectively. By setting SDG quantitation value on X axis and SE-HPLC quantitation value on Y axis, a standard curve was drawn with calculation of the R^2^ value. Plus, purified 146S particles were also used to compare the dispersion degree of the 146S particles in the fractions obtained by the two methods. The antigens were confirmed to be pure by transmission electron microscopy (data not shown).

SE-HPLC was performed on a TSKgel G4000PWXL (300 mm × 7.8 mm I.D.) column (TOSOH Bioscience, Tokyo, Japan) combined with a TSKgel PWXL Guardcol (40 mm × 6.0 mm) guard column (TOSOH Bioscience) using an Agilent 1260 Infinity II system (Agilent Technologies, Santa Clara, CA, USA) composed of a quaternary pump with an online degasser, autosampler with a sample cooler, fraction collector, thermostatic column compartment, and variable wavelength detector operating at 254 nm [13]. The mobile phase was composed of 30 mM Tris-HCl and 400 mM NaCl (pH 8.0), and the flow rate was set at 0.5 mL/min. The area under the target peak was integrated using OpenLAB CDS ChemStation software, and the quantity of 146S particles (µg/mL) was calculated according to a previous study [13]. Time-based fractionation was performed from 6 to 26 min at 2 min intervals.

### 2.4. SDS-PAGE and Western Blot Analysis

Samples were mixed with a 4× lithium dodecyl sulfate sample buffer (Invitrogen, Carlsbad, CA, USA) containing a sample-reducing agent (Invitrogen) and boiled at 95 °C for 10 min. Proteins were separated on 4–12% Bis-Tris gels (Invitrogen). The gel was stained by Coomassie Brilliant Blue R-250 staining solution (Bio-Rad, Hercules, CA, USA) for 1 h with gentle agitation. The stained gel was then treated with a destaining solution (Biosesang, Sungnam, Korea) and agitated until the protein bands were visible.

For Western blotting, the gel was transferred onto a polyvinylidene fluoride membrane (Invitrogen) using an iBlot Gel Transfer Device (Invitrogen). The membranes were blocked with 2% skim milk in phosphate-buffered saline (PBS) containing 0.1% Tween 20 (PBS-T) for 1 h at room temperature (RT) with shaking, washed thrice with PBS-T for 10 min, and then incubated overnight with a home-made primary antibody against FMDV VP1 at 4 °C. The following day, the membranes were washed thrice with PBS-T and incubated with HRP-conjugated goat anti-mouse secondary antibody (Invitrogen) for 1 h at RT. The antibody–antigen complexes were visualized with ECL Western blotting substrate (Amersham, Buckinghamshire, UK) using an Azure C600 device (Azure Biosystems, Dublin, CA, USA).

### 2.5. Stability Tests

Both accelerated stability and long-term preservability tests were conducted to compare the stability of FMDV O BE and BEV. All samples were quantified using SE-HPLC. An accelerated stability tests were conducted by heating the samples to approximately 30 µg/mL at 50 °C for 30 min. The recovery (%) was calculated from the remaining content of the virus particles after heating when the initial particle contents of the two viruses were set to 100%. Meanwhile, a long-term preservability test was performed by maintaining antigens at approximately 30 µg/mL at 4 °C for 3 months. After one month and three months, recovery (%) was calculated from the remaining content of the virus particles after storage when the initial particle contents of the two viruses were set to 100%.

### 2.6. Establishment of Standard Curves Using BEV Standards

As a standard material, BEV pure antigens were prepared at high (154.5 µg/mL by SDG quantitation) and low (15.5 µg/mL by the SDG quantitation) concentrations. The original (1×) samples of high and low concentrations of BEV standards were quantified by SDG once at the time of standard preparation and the concentrations of each diluted sample (1/2× to 1/16×) were arithmetically calculated to be set as the SDG value. Meanwhile, either the high or low concentration of 1× BEV standards was serially diluted two-fold with Tris-KCl buffer (pH 7.6) to 1/16× for FMDV quantitation by SE-HPLC. BEV standards from 1× to 1/16× were all quantified using SE-HPLC to be set as SE-HPLC value. By setting SDG value of BEV standards on X axis and SE-HPLC value of the standards on Y axis, a standard curve was drawn with calculation of the R^2^ value.

### 2.7. Adjusting the Amounts of FMDV Particles Measured by SE-HPLC by a Standard Curve Using BEV

Four FMDV strains (O PA2, O BE, A22 IRQ, and A YC) proliferated in BHK-21 cells and were purified by SDG. The antigens were prepared at high (>30 µg/mL) and low (<5 µg/mL) concentrations for the quantification of 146S particles by SDG and SE-HPLC. With the standard curve derived from BEV standard quantification, the amount of FMDV 146S particles measured by SE-HPLC was adjusted to the SDG values by interpolation to the standard curve. The adjusted SE-HPLC values were compared to the original SDG values to calculate the error rate between the amount of FMDV 146S particles obtained by SDG and HPLC.

### 2.8. Statistical Analysis

All experiments were performed in triplicate, and representative data are presented. In the dot plots, a regression line drawing and the R^2^ value calculation were performed using Microsoft Excel 2016 (Microsoft, Redmond, WA, USA). In the bar graphs, all values are presented as mean ± standard error of the mean. Statistical analyses were performed using a paired *t*-test using SPSS Statistics version 26.0. software (IBM Corp., Armonk, NY, USA). Statistical significance was defined as * *p* < 0.05, or ** *p* < 0.01.

## 3. Results

### 3.1. Comparison of HPLC and SDG for the Detection of 146S Particles

**Figure 1:**
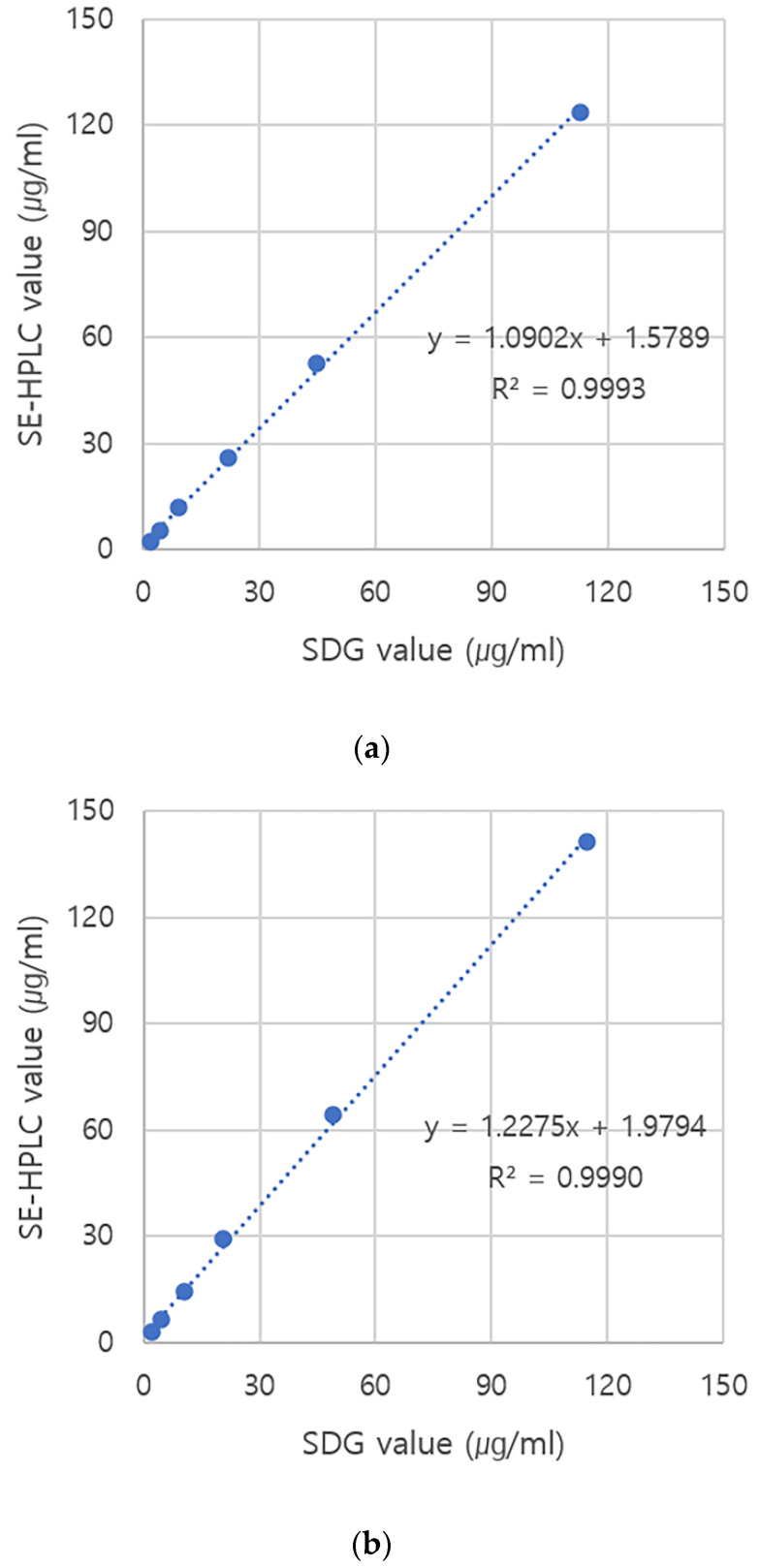

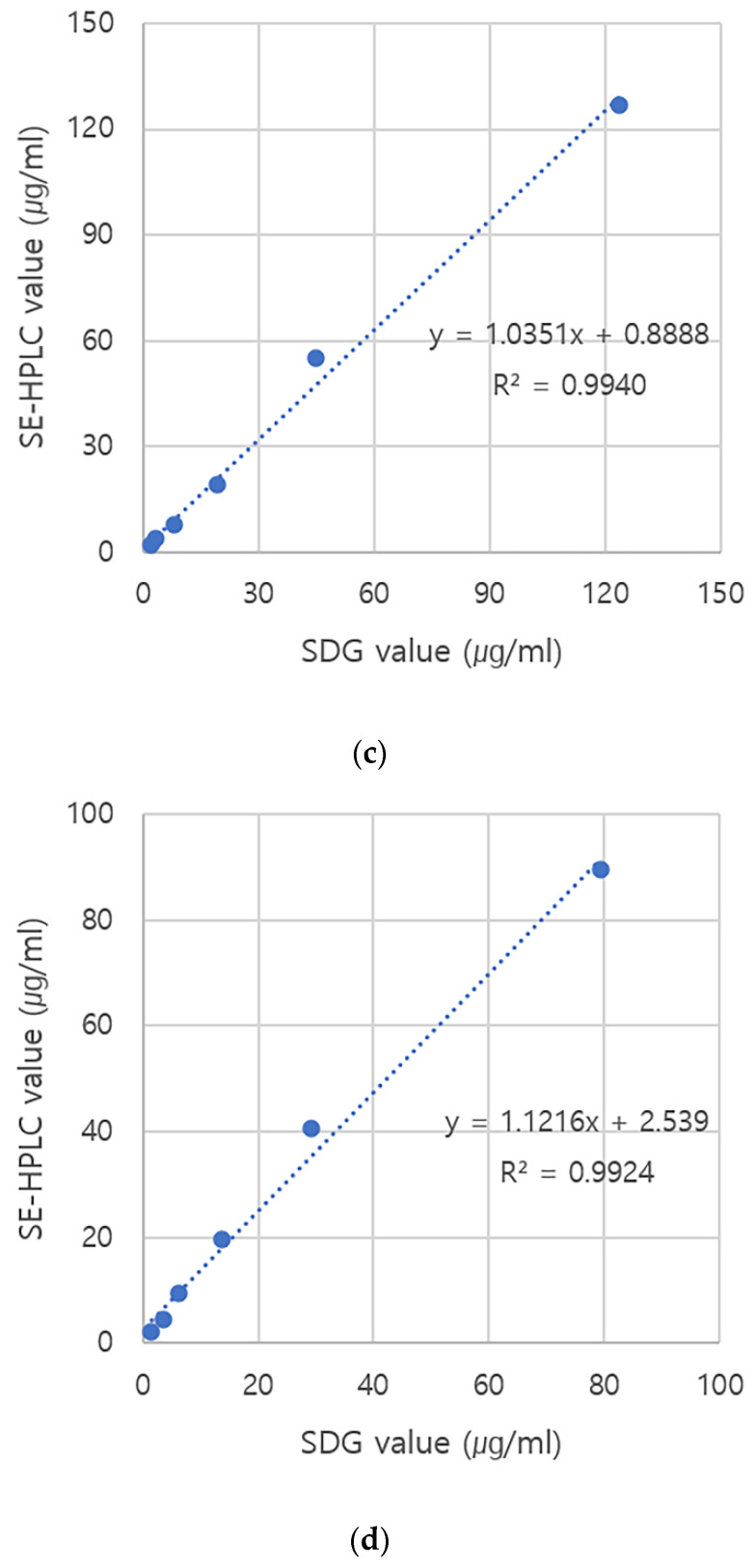
Differences in the amount of 146S particles obtained from SDG and SE-HPLC. (**a**) Correlation between the 146S particle quantity of FMDV O PA2 measured by SDG and that measured by SE-HPLC. (**b**) Correlation between the 146S particle quantity of FMDV O BE measured by SDG and that measured by SE-HPLC. (**c**) Correlation between the 146S particle quantity of FMDV A22 IRQ measured by SDG and that measured by SE-HPLC. (**d**) Correlation between the 146S particle quantity of FMDV A YC measured by SDG and that measured by SE-HPLC. Abbreviations: SDG, sucrose density gradient ultracentrifugation; SE-HPLC, size-exclusion high-performance liquid chromatography.

**Figure 2:**
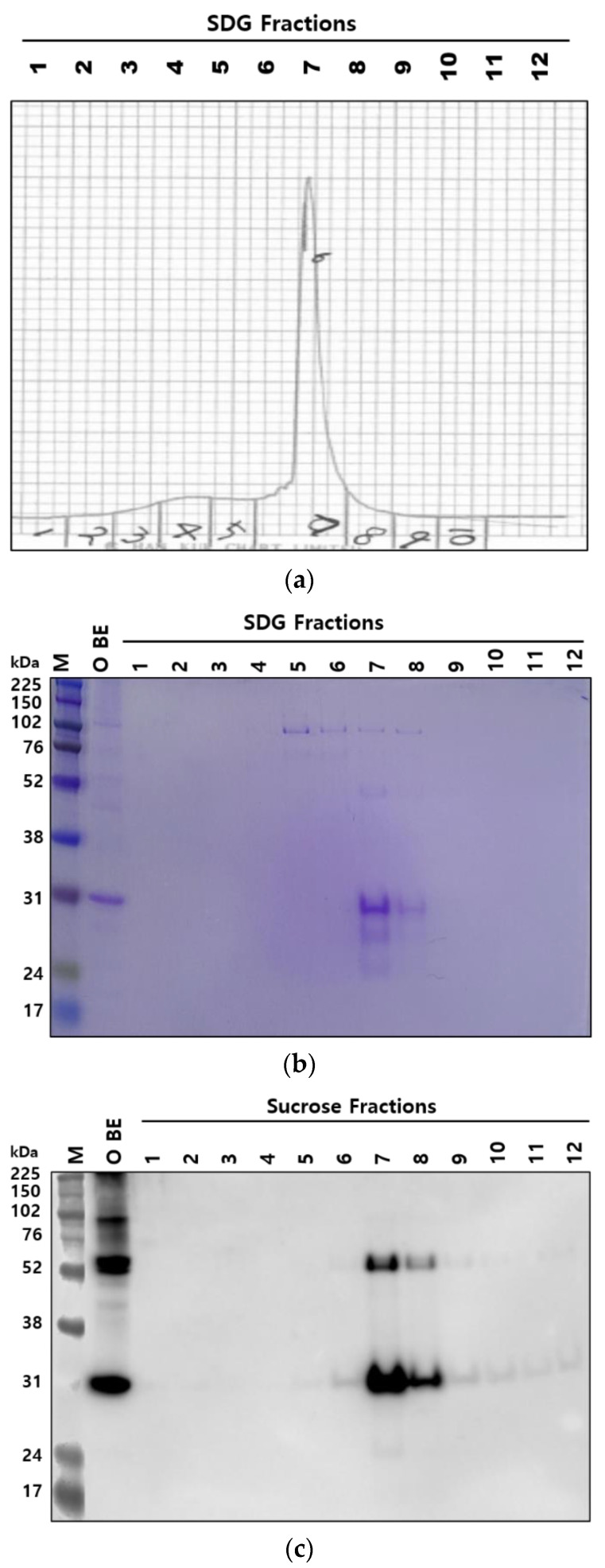

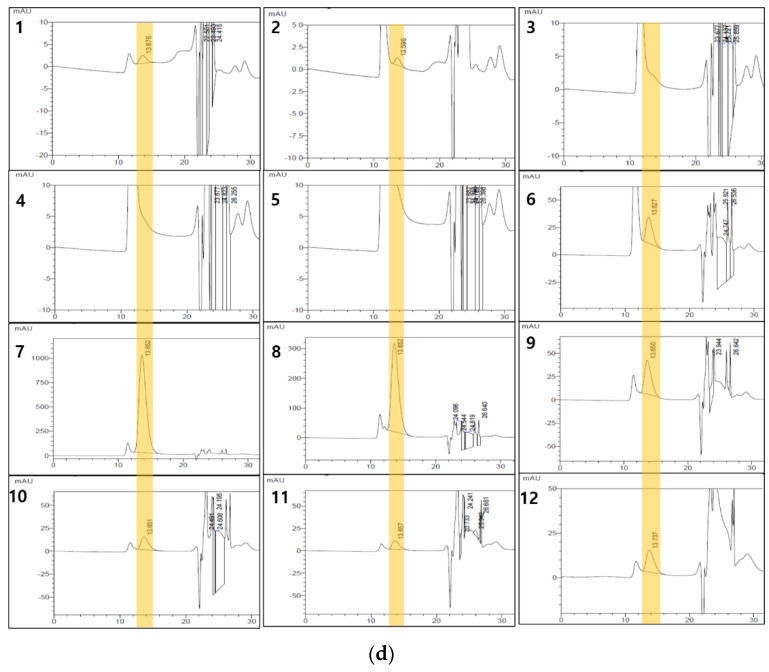
Wide dispersion of vaccine antigens by SDG fractionation. (**a**) An original chromatogram from SDG fractionation of SDG-purified FMDV O BE vaccine antigen. Numbers above the chart indicate each fraction number. (**b**) SDS-PAGE results of each SDG fraction from (**a**). FMDV O BE pure antigen was administered as a positive control. Between 24 and 31 kDa, bands of FMDV structural protein subunits are shown. (**c**) Western blot results of each SDG fraction from (**a**) using the VP1 antibody. VP1 bands were detected at approximately 31 kDa. (**d**) Original chromatograms from SE-HPLC of each SDG fraction from (**a**). Numbers in the top left of each chromatogram indicate each SDG fraction number injected into SE-HPLC. Yellow backgrounds indicate the detection time of the target peak. Abbreviations: SDG, sucrose density gradient ultracentrifugation; SE-HPLC, size-exclusion high-performance liquid chromatography.

**Figure 3:**
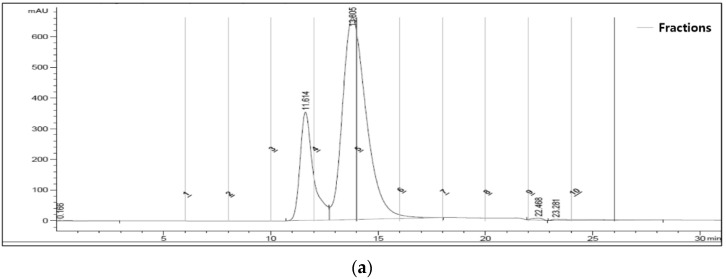

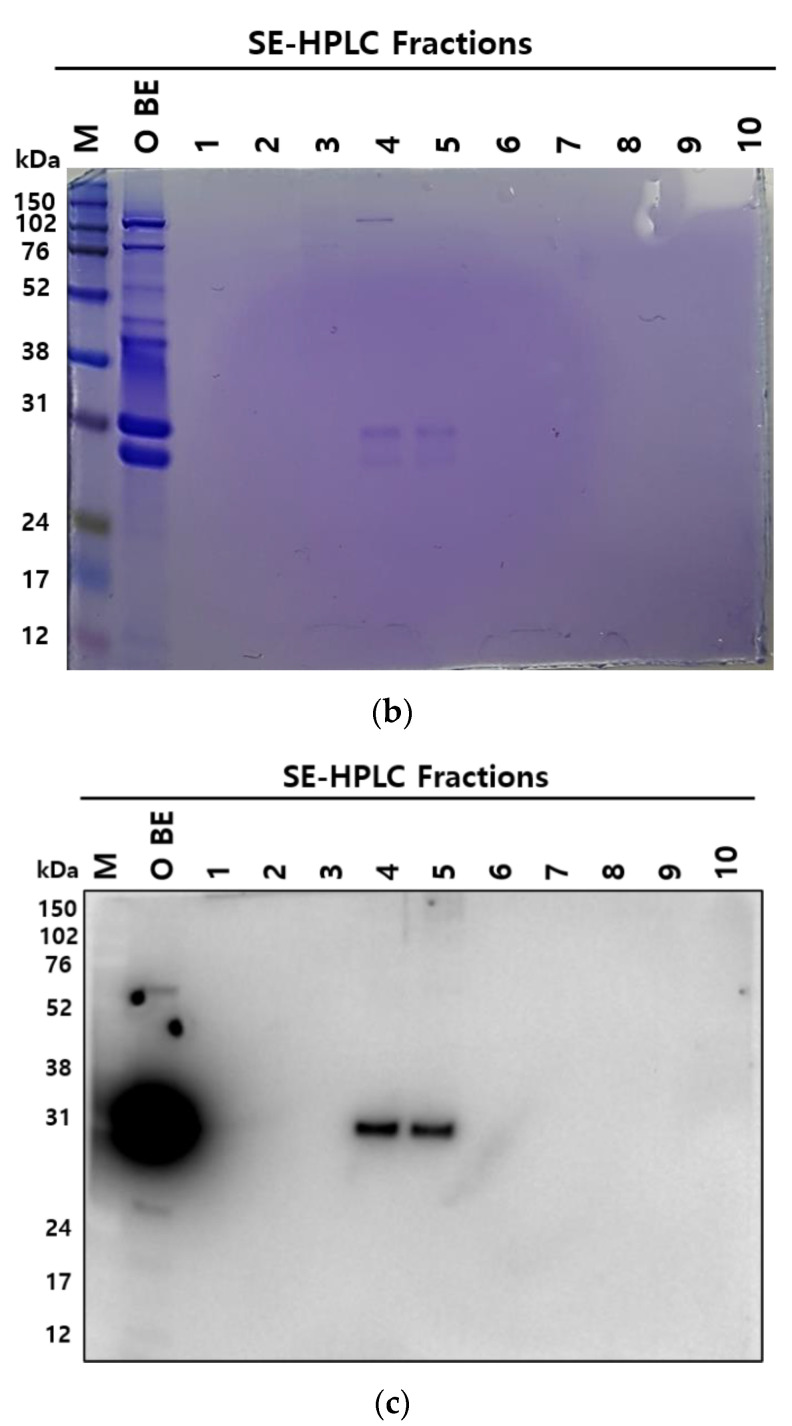
Narrow dispersion of vaccine antigens by SE-HPLC fractionation. (**a**) An original chromatogram from SE-HPLC fractionation of SDG-purified FMDV O BE vaccine antigen. Numbers underlined at the gray vertical lines indicate the starting point of each fraction number. (**b**) SDS-PAGE results of each SE-HPLC fraction from (**a**). FMDV O BE pure antigen was administered as a positive control. Between 24 and 31 kDa, bands of FMDV structural protein subunits are shown. (**c**) Western blot results of each SE-HPLC fraction from (**a**) using the VP1 antibody. VP1 bands were detected at approximately 31 kDa. Abbreviations: SDG, sucrose density gradient ultracentrifugation; SE-HPLC, size-exclusion high-performance liquid chromatography.

The purified 146S particles of four FMDV strains (O PA2, O BE, A22 IRQ, and A YC) were quantified by SDG and SE-HPLC. The same samples were loaded, however, there were differences between the amount of 146S particles obtained from the two quantitation methods. Although there was a close correlation (R^2^ value > 0.99) between the two methods, the quantity measured by SE-HPLC was generally higher than that measured by the SDG for all tested FMDV strains (Figure 1). The fractions obtained from FMDV O BE by SDG and SE-HPLC were analyzed by SDS-PAGE and Western blotting to investigate why the amount of 146S particles measured by SE-HPLC differed from that calculated by SDG. The O BE was purified and fractionated into 12 tubes using SDG (Figure 2a). As expected, the peaks in fractions 7 and 8 corresponded to the 146S particles. SDS-PAGE exhibited a positive band only in fractions 7 and 8 (Figure 2b); however, Western blotting showed positive reactions in fractions 7 and 8 and in other fractions (Figure 2c). In addition, when the SDG fractions were reanalyzed by SE-HPLC, they exhibited peaks corresponding to 146S particles in fractions 6–12 (Figure 2d). Small peaks were also observed for fractions 1 and 2. In contrast, out of 10 fractions, fractions 4 and 5 showed the 146S peak at approximately 13.8 min when the samples were analyzed by SE-HPLC (Figure 3a). SDS-PAGE (Figure 3b) and Western blotting (Figure 3c) also exhibited the same results as those of the chromatogram, such that the positive bands were only observed in fractions 4 and 5. The peak in fraction 3 was unrelated to the 146S particle because it did not react with the antibody against VP1 (Figure 3c).

### 3.2. Assessment of BEV as a Surrogate Standard for FMDV

**Figure 4:**
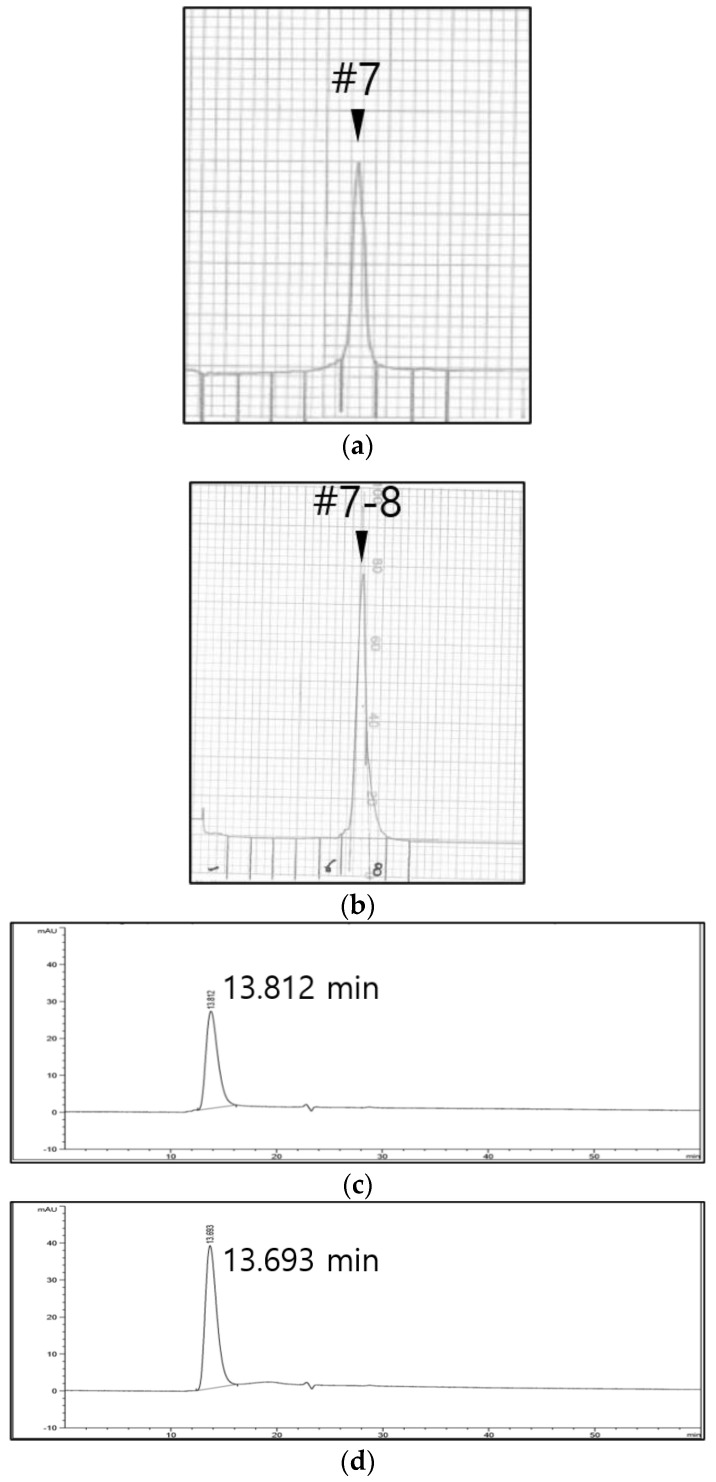
Physical characteristics of BEV as an FMDV surrogate for quantification. (**a**) An original chromatogram from SDG fractionation of SDG-purified FMDV O BE vaccine antigen. Numbers above the target peak indicate the main fraction number. (**b**) An original chromatogram from SDG fractionation of SDG-purified BEV antigen. Numbers above the target peak indicate the main fraction number. (**c**) An original chromatogram from SE-HPLC fractionation of SDG-purified FMDV O BE vaccine antigen. Detection time of the target peak was noted. (**d**) An original chromatogram from SE-HPLC fractionation of SDG-purified BEV antigen. Detection time of the target peak was noted. Abbreviations: BEV, bovine enterovirus; SDG, sucrose density gradient ultracentrifugation; SE-HPLC, size-exclusion high-performance liquid chromatography.

**Figure 5:**
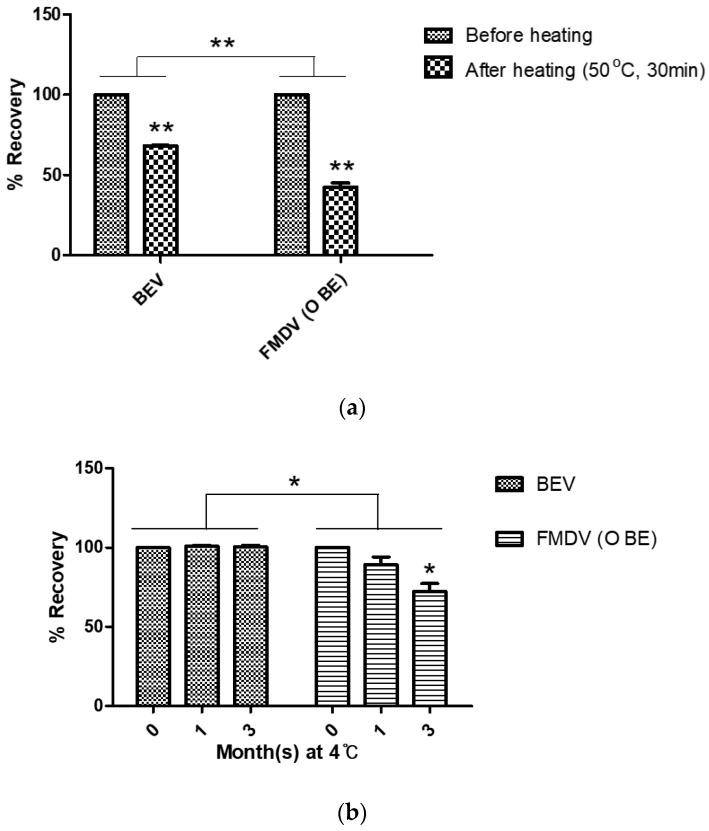
Stability of BEV as an FMDV surrogate for quantification standard. (**a**) The accelerated stability test results by heating at 50 °C for 30 min. (**b**) The long-term preservability test results by maintaining each viral antigen at 4 °C for 3 months. Initial content before heating or storage was set as 100%. * *p* < 0.05, ** *p* < 0.01. Abbreviations: BEV, bovine enterovirus.

A stable standard virus was required to investigate the correlation between the quantity of FMDV 146S particles measured by SDG and SE-HPLC. FMDV is generally unstable compared to other viruses; therefore, BEV, a stable picornavirus, was employed as a standard material for the quantification of virus particles. BEV proliferated in BHK-21 cells and was purified in the same manner as that for FMDV. The peak fractions of BEV by SDG and HPLC were almost the same as those of O BE (Figure 4). As expected, BEV was significantly more stable than the O BE in both an accelerated test at 50 °C for 30 min (Figure 5a) and a long-term preservability test for 3 months at 4 °C (Figure 5b).

### 3.3. Establishment of Standard Curves Using BEV Standards

**Figure 6:**
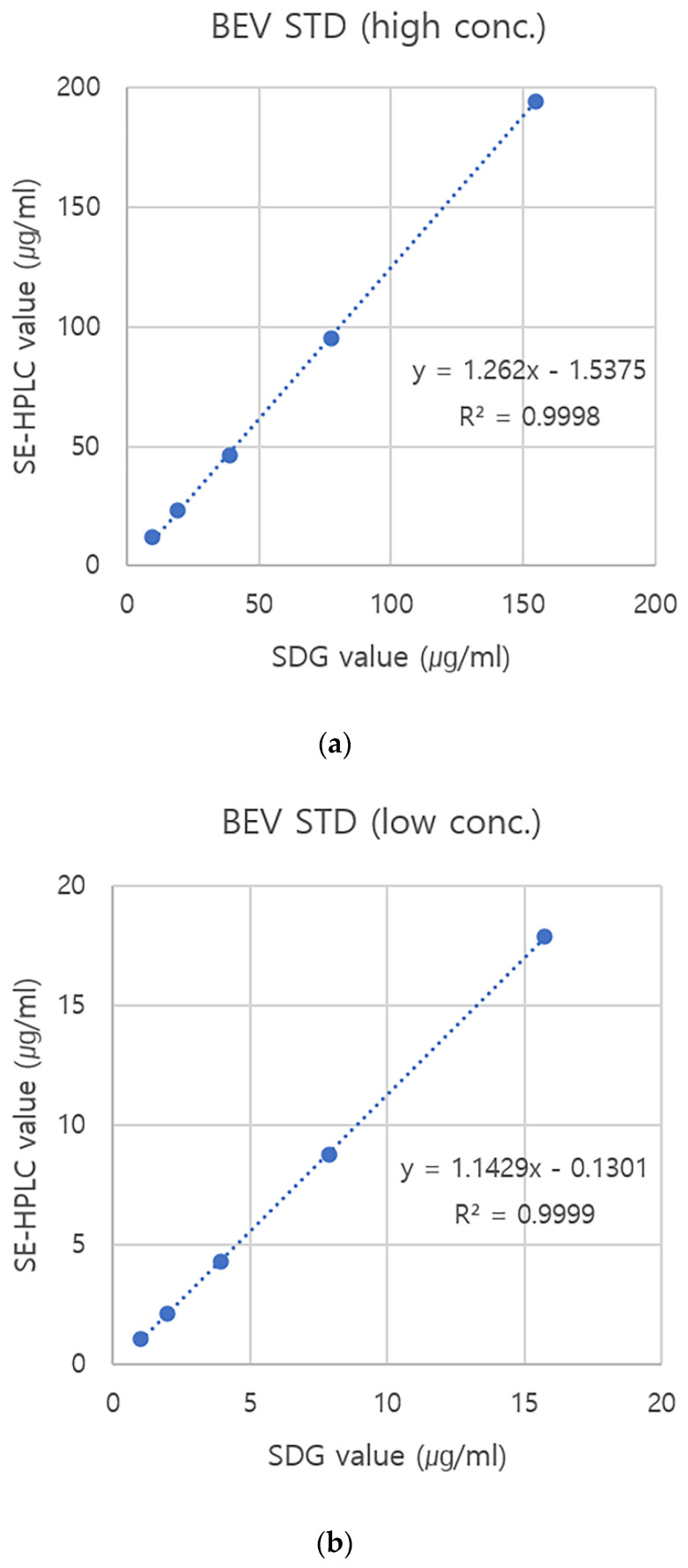

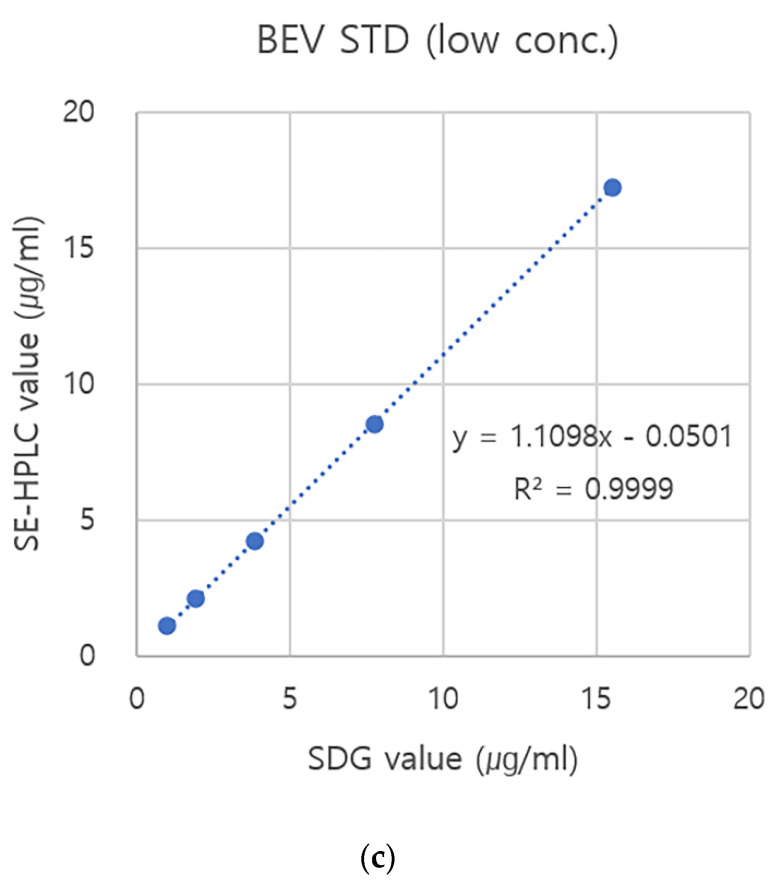
BEV standard curves showing correlation between the quantitation value of SE-HPLC and that of SDG. (**a**) A BEV standard curve at high concentration used in adjustment of FMD vaccine antigen content at a high concentration of FMDV pure antigens. (**b**) A BEV standard curve at low concentration used in adjustment of FMD vaccine antigen content at a low concentration of FMDV pure antigens. (**c**) A BEV standard curve at low concentration used in adjustment of FMD vaccine antigen content in a crude virus infection supernatant. Abbreviations: BEV, bovine enterovirus; STD, standard; SDG, sucrose density gradient ultracentrifugation; SE-HPLC, size-exclusion high-performance liquid chromatography.

BEV standards, prepared at a high concentration and quantified by SDG at the time of preparation, were serially diluted and quantified by the SE-HPLC in case the high concentration (>15 µg/mL) of FMDV samples were quantified by SE-HPLC. A standard curve was drawn from the correlation between SDG value and SE-HPLC value. Similar to the case of FMDV quantitation, there were differences in the amount of BEV standards obtained from the two quantification methods. Although there was close correlation (R^2^ value = 0.9998) between the two methods, the quantity measured by SE-HPLC was higher than that measured by SDG (Figure 6a). Meanwhile, BEV standards at a low concentration were also serially diluted and quantified by the SE-HPLC in case an FMDV crude virus infection supernatant (CVIS) or other low concentrations (below 15 µg/mL) of FMDV samples were quantified by SE-HPLC. Standard curves were drawn from the correlation between the SDG value and SE-HPLC value. A BEV standard curve at a low concentration, drawn for the quantitation of a low concentration of purified FMD vaccine antigens, exhibited a close correlation (R^2^ value = 0.9999) between the two methods; however, the amount calculated by SE-HPLC was also higher than that calculated by SDG (Figure 6b). The other BEV standard curve at a low concentration, drawn for the quantitation of FMDV CVIS, also exhibited a close correlation (R^2^ value = 0.9999) between the two methods, although the quantity obtained from SE-HPLC was higher than that obtained from SDG (Figure 6c).

### 3.4. Comparative Quantification of FMDV Particles between the SDG and SE-HPLC and Decrease of the Gap Using a BEV Standard

Four types of pure FMDV particles were prepared at high and low concentrations and quantified using SDG and SE-HPLC. The difference between the two quantitative methods was corrected by interpolating the quantity measured by SE-HPLC into a standard curve established based on the BEV standard. The correction formula is as follows: Adjusted SDG value = (SE-HPLC value + 1.5375) ÷ 1.262 (Figure 6a). When the purified FMD vaccine antigen (high concentration) was applied, the absolute quantitation error rate between the two quantification methods ranged from 19.13% to 30.40% for all four FMDV types. However, according to the corrected values derived from the standard curve prepared using the BEV standard (high concentration), the error rate between the two quantification methods converged to within ±10% (Figure 6a and Table 1). When the purified FMD vaccine antigen (low concentration) was applied, the absolute quantitation error rate between the two quantification methods ranged from 19.77% to 20.49% for all four FMDV types. The error rate between the two quantitative methods converged to within ±10%, based on the corrected values derived from the standard curve prepared by using the BEV standard (low concentration). The correction formula used in this experimental set was as follows: Adjusted SDG value = (SE-HPLC value + 0.1301) ÷ 1.1429 (Figure 6b and Table 1). When the crude virus infection supernatant was applied with the previously reported optimal pretreatment method [12], the absolute quantitation error rate between the two quantification methods ranged from 15.03% to 19.14% for all four FMDV types. The error rate between the two quantitative methods converged to within ± 10%, based on the corrected values derived from the standard curve prepared using the BEV standard. The correction formula used in this experimental set was as follows: Adjusted SDG value = (SE-HPLC value + 0.0501) ÷ 1.1098 (Figure 6c and Table 2).

## 4. Discussion

SDG is the most widely used method for FMDV 146S particle quantification. It is very complex, requires multiple preparation steps, and is time-consuming [12]. Although a double antibody sandwich enzyme-linked immunosorbent assay (ELISA) was reported to be useful for the quantification of 146S [22], the ELISA is frequently influenced by the preparation procedure and possible nonspecific or incomplete binding, limiting the accuracy of quantification [23]. Therefore, a simple SE-HPLC method that can overcome these problems has been established [12,13,24]. SE-HPLC was also employed to quantify other picornaviruses [23,24].

However, when the above SDG and SE-HPLC methods were employed for the quantification of the same FMDV samples, the value calculated by SE-HPLC was likely to be slightly higher than the value calculated by SDG. This phenomenon was also reported in a previous study [13]. However, the cause is unknown; therefore, this study aimed to identify the reason for the disparity and provide a solution to offset the difference between the two methods by employing a standard curve using BEV as a stable surrogate virus.

First, the FMDV O BE isolated at the time of the FMD outbreak in Korea was propagated and purified using SDG. These purified virus particles were applied to SDG and SE-HPLC for quantification. In SDG, FMDV structural proteins (VP1, 2, and 3) were identified in the peak fraction, and the pattern of the structural protein band was the same as that previously reported [14]. However, Western blotting confirmed the presence of the VP1 protein in fractions other than the target peak using an antibody against the VP1. In principle, since the purified FMDV was applied to SDG, the VP1 protein should be detected only in peak fractions 7 and 8. Considering that the VP1 protein was also detected in other fractions, the 146S particles was widely dispersed over the peak fractions, indicating that the SDG may underestimate the 146S content in the sample because we only calculated the 146S particles in peak fractions. To confirm whether the VP1 band observed in the fractions other than the peak was derived from broken virus particles, the fractions were subjected to SE-HPLC. The peak corresponding to the 146S particle was observed in peak fractions and other neighboring fractions. All peaks were detected at approximately 14 min, indicating the presence of 146S particles. If the peak was derived from 12S, the degraded form from the whole virus particle, it should have been detected at approximately 17 min [14].

Alternatively, when the same purified antigen was applied to SE-HPLC, structural protein bands were observed only in the target peak fractions and not in fractions other than the peak by SDS-PAGE and Western blotting, indicating that the virus particles in the sample were correctly measured by SE-HPLC, not dispersed as shown in the SDG.

SDG has been used as a reliable method for the quantification of 146S particles over the past four decades, although it has a technical burden, time-consuming procedures, and poor repeatability [13,25]. Instead, SE-HPLC is rapid, convenient and easy to standardize and automate, although it requires the pretreatment of unpurified samples to remove nucleic acids, causing severe interference, and there is a potential concern that 146S and 75S particles cannot be discriminated by SE-HPLC alone [13,14,25].

In this regard, SE-HPLC is superior to SDG in various aspects for measuring the quantity of FMDV particles [13,14]. However, this is the first report to provide experimental evidence of the difference between SDG and SE-HPLC for the quantification of the FMDV particles.

Numerous studies were performed using SDG for the quantification of FMDV particles; therefore, it would be convenient if the values measured by SE-HPLC were changed to the values measured by SDG. Therefore, a standard curve using a surrogate virus that has the same size and density as that of the FMDV, but is more stable, was devised. BEV of the picornavirus family is generally considered to be non-virulent or of low virulence, not highly pathogenic, and resistant to acidic environments, making it a good candidate for a vaccine vector [26]. As shown in Figure 4, when the virus was purified by SDG and SE-HPLC, a peak of virus particles was observed at the same location as that of the FMDV. As storage stability is important for the use of a standard material, heat stability tests at 50 °C and long-term preservability tests at 4 °C were performed, and as expected, the BEV was far more stable than the FMDV (Figure 5).

After measuring the quantity of the BEV standard by SDG, the standard samples prepared by serial dilution were measured by SE-HPLC, a standard curve was drawn, and the SDG value was predicted by interpolating the SE-HPLC value into the standard curve for the unknown samples. For the four FMD viruses, the difference between the value measured by SDG and the value measured by SE-HPLC showed a difference of approximately >20% regardless of the virus concentration. When comparing the adjusted value calculated by interpolating the SE-HPLC value into the standard curve with the initial SDG value, the difference was reduced to <10%. Normally, the accuracy of the analytical method is set within 15% for the samples [13,16,27]. Even for the crude virus infection supernatant, which is an unpurified sample, a deviation of >15% was reduced to <10% using the same method incorporating the standard curve.

Moreover, BEV standards are stable and can be produced easily in biosafety level 2 facilities in contrast to FMDV, for which stability is low and requires biosafety level 3 facilities for production. Therefore, these standards not only can be used to adjust the SE-HPLC quantitation value to the SDG quantitation value, but they also can be applied to calibrate and validate analytical instruments for the quantification of FMDV.

## 5. Conclusions

It is important to be able to predict the quantitative value by SDG using the values measured by SE-HPLC for unknown samples using a quantification standard, because it is impossible to match the values between the SDG and SE-HPLC by a simple multiplication of the constant. Although further studies are required to discriminate 75S particles from 146S particles by SE-HPLC, this result would be useful to researchers addressing FMDV for the quantification of FMDV by SE-HPLC [28].

## Figures and Tables

**Table 1:** Adjusting the 146S antigen quantitation value of SE-HPLC to that of SDG using the BEV standard: applied to high and low concentrations of purified vaccine antigens.

Purified Ag	Strain	Practical Value (µg/mL)		Adjusted Value: HPLC to SDG	% Error Rate (HPLC/SDG)	% Error Rate (Adjusted Value/SDG)
		SDG	HPLC			
High conc.	O PA2	45.71	58.62	47.67	28.26	4.30
High conc.	O BE	48.58	60.61	49.25	24.77	1.37
High conc.	A22 IRQ	32.46	42.32	34.52	30.40	6.35
High conc.	A YC	37.76	44.98	36.86	19.13	−2.37
Low conc.	O PA2	3.28	3.94	3.56	20.19	8.63
Low conc.	O BE	3.27	3.93	3.56	20.28	8.72
Low conc.	A22 IRQ	3.77	4.54	4.09	20.49	8.44
Low conc.	A YC	3.97	4.75	4.27	19.77	7.66

Abbreviations: SDG, sucrose density gradient ultracentrifugation; SE-HPLC, size-exclusion high-performance liquid chromatography.

**Table 2:** Adjusting the 146S antigen value of SE-HPLC to that of SDG using the BEV standard: applied to a crude virus infection supernatant (CVIS).

CVIS	Practical Value (µg/mL)		Adjusted Value (µg/mL): HPLC to SDG	% Error Rate (HPLC/SDG)	% Error Rate (Adjusted Value/SDG)
Strain	SDG	HPLC			
O PA2	1.85	2.13	1.96	15.04	6.10
O BE	4.61	5.30	4.82	15.03	4.63
A22 IRQ	3.94	4.64	4.22	17.66	7.16
A YC	2.20	2.62	2.41	19.14	9.41

Abbreviations: SDG, sucrose density gradient ultracentrifugation; SE-HPLC, size-exclusion high-performance liquid chromatography.

## Data Availability

Not applicable.
